# Catalytic Transformation of Nitroarenes to Amines over Ba_(1−x)_Sr_x_TiO_3_ (0 < x < 1) Perovskites in Water

**DOI:** 10.3390/molecules29071416

**Published:** 2024-03-22

**Authors:** Iwona Kuźniarska-Biernacka, Barbara Garbarz-Glos, Elżbieta Skiba, Waldemar Maniukiewicz, Marta Monteiro, Wojciech Bąk, Dariusz Szydłowski, Cristina Freire

**Affiliations:** 1 REQUIMTE/LAQV, Departamento de Química e Bioquímica, Faculdade de Ciencias, Universidade do Porto, Rua do Campo Alegre s/n, 4169-007 Porto, Portugal; marta_monteiro11@hotmail.com (M.M.); acfreire@fc.up.pt (C.F.); 2 Institute of Technology, University of the National Education Commission, Podchorążych 2, 30-084 Kraków, Poland; wojciech.bak@up.krakow.pl; 3 Institute of General and Ecological Chemistry, Lodz University of Technology, Żeromskiego 116, 90-924 Łódź, Poland; elzbieta.skiba@p.lodz.pl (E.S.); waldemar.maniukiewicz@p.lodz.pl (W.M.); 4 Evidence Law and Forensic Technology, University of the National Education Commission, Podchorążych 2, 30-084 Kraków, Poland; dariusz.szydlowski@up.krakow.pl

**Keywords:** perovskite, catalysis, valorization of toxic pollutant, dielectric properties

## Abstract

This work is focused on the application of lanthanide-free perovskite Ba_1−x_Sr_x_TiO_3_ (0 < x < 1) in valorization of toxic pollutants as 4-nitrophenol (4-NPh). The series of perovskites were fabricated by facile, one-step solid-state preparation method and characterized via various techniques: elemental analysis (Inductively Coupled Plasma Optical Emission Spectrometry, ICP-OES), powder X-ray diffraction (XRD), scanning electron microscopy (SEM), Fourier-transform infrared spectroscopy (FTIR) and dielectric properties (impedance spectroscopy, IS). The methods confirmed the assumed composition, structure and high purity of the materials. The results showed that substitution of Ba^2+^ by Sr^2+^ in the perovskite crystal lattice influenced the dielectric properties of samples and the size of the grains. The absorption and catalytic properties of Ba_(1−x)_Sr_x_TiO_3_ (0 < x < 1) series were evaluated in reduction of 4-NPh in water using NaBH_4_ as reducing agent. No adsorption of 4-NPh was found for all the materials during 180 min of contact (experiment without reducing agent), and the best catalytic performance was found for the Ba_(1−x)_Sr_x_TiO_3_ (x = 0.3) sample. The catalytic transformation of 4-NPh to 4-APh follows a pseudo-first-order model, and the catalysts can be easily regenerated via mild annealing (300 °C).

## 1. Introduction

4-nitrophenol (4-NPh) has been identified by the US Environmental Protection Agency (EPA) as one of the priority pollutants due to its danger and toxicity. It is widely used in a variety of industries (paper, textile and pharmaceutics), and it is highly soluble and stable in water.

A variety of techniques have been developed for 4-NPh removal from contaminated water bodies including adsorption [1], advanced oxidation [2,3,4] and reduction [5,6]. Among them, reduction to 4-aminophenol (4-APh) is of interest, as less toxic 4-APh has commercial relevance as an important intermediate for synthesis, making the process interesting also from the industrial point of view. It has been demonstrated that nanocatalysts incorporating noble metals such as Pt, Ag or Au, whether as mono- or bi-metallic nanoparticles (NPs), supported on a variety of materials including organic polymers, silicas, organic–inorganic hybrid assemblies and biological matrices, exhibit remarkable activity in the reduction of 4-nitrophenol (4-NPh) in the presence of sodium borohydride (NaBH_4_) as a reducing agent. This catalytic process achieved complete reduction of 4-NPh within a short time (ranging from 0.5 to 30 min) [5,7]. However, the high costs of noble metals and constant, reliable supply restrict their utilization. Thus, alternative, cheaper, highly active and stable catalysts are needed. Noble-metal-free multicomponent composites containing transition metals (Cu, Fe, Ni or Mn) or their oxides including ferrites and minerals (hydroxyapatite) [8], incorporated into metal–organic framework (MOF) networks [9] or reduced graphene sheets [10] with excellent catalytic performance towards reduction of 4-NPh (total conversion in less than 2 min), have achieved more attention due to their availability. But overall cost of their preparation (harsh conditions, high pH > 10, organic solvents and chemical oxidation/reduction intermediate steps) and difficulty in reproducibility of the catalyst’s preparation (distribution and loading of active sites in nanocomposites) and problems with scale-up of the fabrication process make their practical application difficult. Perovskite-type mixed oxides with a stoichiometric ABO_3_, (where A and B are different cations) are a well-known class of crystalline metal oxides. Due to the presence of various metals in their structures and intrinsic crystal lattice defects, they possess the capacity to demonstrate significant catalytic potential when utilized as heterogeneous catalysts.

In recent years, those consisting of rare earth and 3d transition metals are of interest and were used as a catalyst in various processes: oxygen reduction reaction [11], oxidation of toxic chlorinated aromatics [12] and decomposition of NO [13] among others. Also, perovskite (LaMnO_3_) encapsulated into MOF-5 (containing Zn and terephthalic acid) pores was used as an alternative for noble metal catalysts in hydrogenation of nitroarenes [14]; the catalyst was able transform 80% of 4-NPh into 4-APh in 5 min. Similarly, to noble metals, the high price and scarcity of the lanthanides are the main problems that have hindered their large-scale industrial application. Additionally, these metals are sensitive to moisture and air when they are in complex forms [15]. Due to that, the perovskites containing alkaline earth metals, namely ferroelectric barium strontium titanate (Ba_(1−x)_Sr_x_TiO_3_), widely applied as super capacitors, dielectrics, ceramics and (photo)catalysts [16], seem a good alternative. 

Here, for the first time, we present the systematic study of catalytic activity of cost-effective Ba_(1−x)_Sr_x_TiO_3_ perovskite series (0 < x < 1) towards the reduction of 4-NPh as an alternative to noble and lanthanide metal catalysts. The perovskite materials Ba_(1−x)_Sr_x_TiO_3_ (1 < x < 0) were prepared by one-step thermal synthesis using different proportions of Ba, Sr and Ti precursors. denoted as BTO, BST_80, BST_70, BST_55, BST_50, BST_40, BST_1 and STO, respectively, for x = 0.0, 0.2, 0.3, 0.45, 0.5, 0.6, 0.99 and 1.0. The materials were characterized by standard methods. The correlation of their chemical composition, structure, morphology and dielectric properties with catalytic activity were discussed.

## 2. Results and Discussion

### 2.1. Characterization of the Ceramics

To ensure the elemental composition of Ba_(1−x)_Sr_x_TiO_3_ (0 < x < 1) samples obtained via solid-phase reaction, a quantitative analysis of the major elements (Ba, Sr and Ti) was performed by ICP-OES. The calculated and found contents of barium, strontium and titanium together with the respective standard deviation of the mean (SE) are summarized in Table 1. The SE values were calculated for three replicates (independent measurements). The low values of SE (ranging from 0.12% to 1.24%) and an excellent concordance between the calculated and found content of elements clearly confirm the assumed composition and high purity of the prepared materials. 

These materials are stable in air and do not change their stoichiometric composition over time (up to 5 months). 

**Figure 1:**
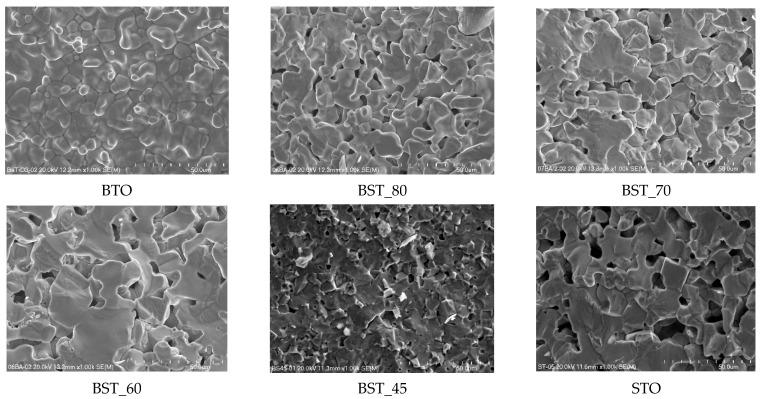
SEM images of the selected of the Ba_(1−x)_Sr_x_TiO_3_ ceramics (magnification 1000×).

The SEM images of fractures for selected Ba_(1−x)_Sr_x_TiO_3_ samples are presented in Figure 1. The microphotographs clearly show that the surface of fracture runs both across grains and along intergranular boundaries. The analysis indicates that the strontium incorporation affects the size of the grains, with a tendency to decrease. The average grain size is in the range of 12–9 μm, excluding the BTO sample, where the grains are larger (15 μm).

**Figure 2:**
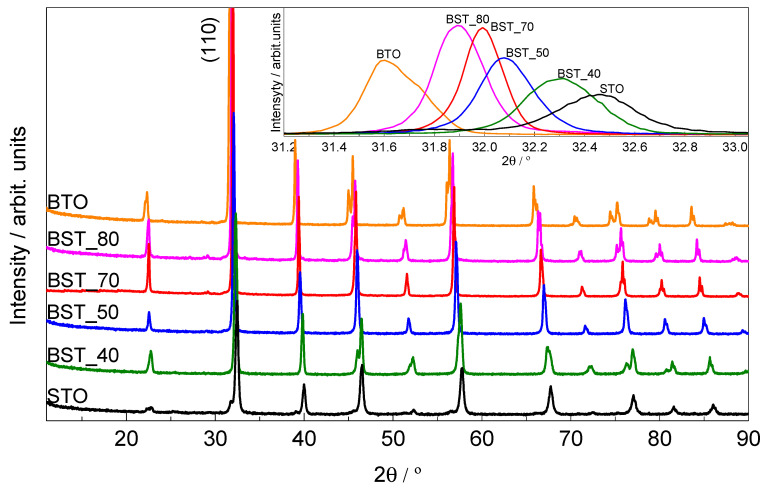
XRD patterns of Ba_(1−x)_Sr_x_TiO_3_ ceramics in 2θ range of 15.0–90.0°, insert, shift of (110) diffraction peak in 2θ range of 31.2–33.0°.

**Figure 3:**
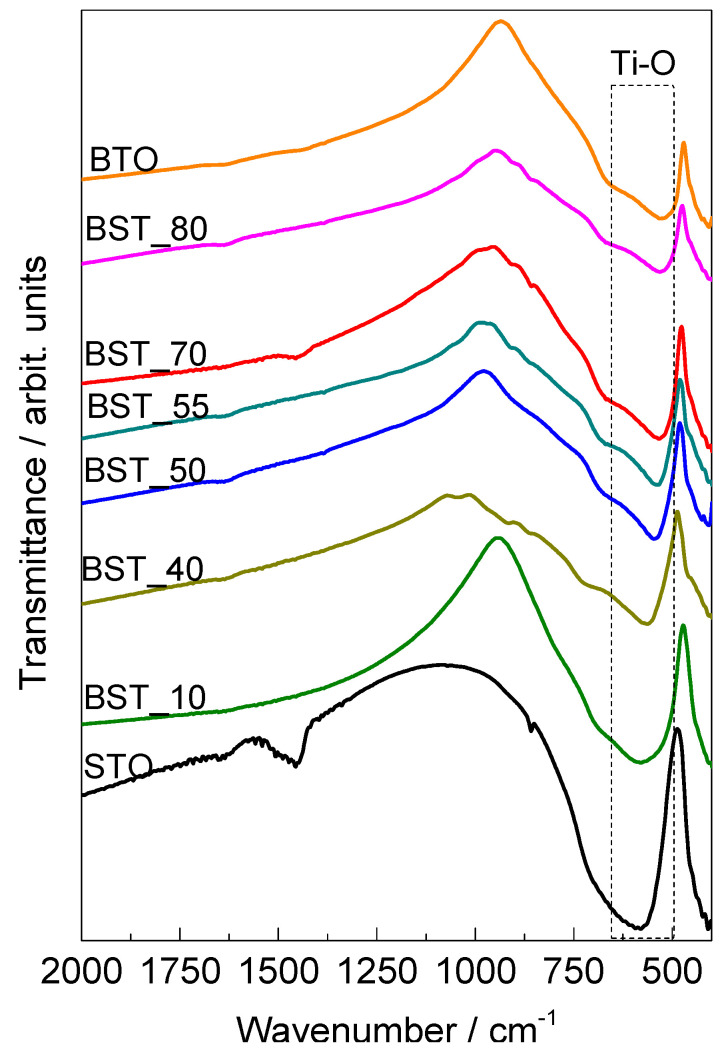
FTIR spectra of Ba_(1−x)_Sr_x_TiO_3_ samples in the region of 500-2000 cm^−1^.

The XRD study shows that all samples crystallize in the pure perovskite phase (Figure 2). Well-resolved peaks in the BTO diffractogram showed good crystallinity of the sample. The BTO diffraction pattern in the 2θ regions of 44–46° shows the splitting the diffraction peaks, this confirms the presence of the tetragonal BaTiO_3_ phase crystallizing in the P4mm space group (JCPDS data no. 05-0626). Moreover, with increasing strontium content in the studied BST samples, the main diffraction peak (110) shifted towards the higher 2θ angles (Figure 3); this indicates a decrease in the values of the unit cell parameters. The change in lattice parameters compared to that of BTO are due to the differences in the ionic radii: r(Ba^2+^) = 1.61 Å, r(Sr^2+^) = 1.31 Å [17]. This fact confirms that the Sr^2+^ ions are substituting Ba^2+^ ions in the lattice of investigated solid solutions. 

FTIR spectra of Ba_(1−x)_Sr_x_TiO_3_ ceramics in the range of 400–2000 cm^−1^ are presented in Figure 3. All the materials show a band within the range of 500 cm^−1^ to 650 cm^−1^ associated with the Ti−O stretching vibration [18]. This band is shifted to higher energy as the strontium content in the samples is increased: BTO (525 cm^−1^), BST_80 (532 cm^−1^), BST_70 (534 cm^−1^), BST_55 (536 cm^−1^), BST_50 (545 cm^−1^), BST_40 (561 cm^−1^), BST_10 (570 cm^−1^) and STO (582 cm^−1^). This shift towards higher energy values is associated with the reduction in lattice parameters. 

As the Sr^2+^ concentration increases, crystal volume decreases, which contracts the distance between Ti^4+^ and O^2−^. As a result, bond strength increases, which enhances the average force constant. These measured Ti-O bond length values fit well with the acquired results from the XRD.

These trends are in agreement with the results presented in the literature for Ba_(1−x)_Sr_x_TiO_3_ (0 ≤ x ≤ 1) samples synthesized using the conventional solid-state reaction technique [18], and for BTO, BST_50 and STO tin films [19], and they were associated with structural discrepancies.

### 2.2. Dielectric Properties

**Figure 4:**
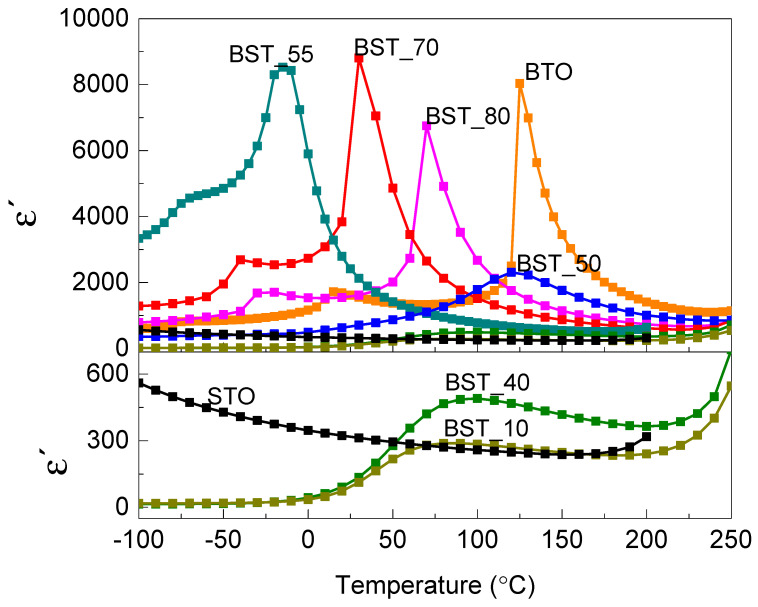
The real part of electric permittivity as a function of temperature for the frequency of measuring field of 1 kHz.

The performed dielectric tests (Figure 4) show that strontium concentration significantly affects the phase transformation temperature as well as having a significant impact on the value of electrical permittivity. For Ba_(1−x)_Sr_x_TiO_3_ samples with substitution of barium ions up to x = 0.3 (BST_70 sample), the electric permittivity peak is sharp (Figure 4). For higher strontium content, the phase transition is broadened over a large temperature range, and the peak dielectric permittivity is suppressed [20], which can be seen for the BST_55 sample (Figure 4). 

The relaxor behaviour is characteristic of samples BST_50, BST_40 and BST_10 and is related to the formation and reorientation of polar nanoregions (PNRs) [21].

In the investigated materials, the dielectric properties, structure and microstructure are closely related [22]. The partial substitution of Ba^2+^ by Sr^2+^ ions causes the transformation of crystalline structures from tetragonal to cubic system.

For the samples with lower concertation of Sr (BST_70 and BST_80), solid solution samples have a tetragonal structure with *P4mm*; however, samples with higher strontium content transformed into a cubic structure with the $Pm\bar{3}m$ space group.

Because the solid solutions of strontium titanate in barium titanate were received as a result of a high-temperature solid-state reaction, each of the grains is surrounded by randomly oriented neighbouring grains, separated by a grain boundary and adhering to it closely [23]. 

The behaviour of the tested materials can be explained by the formation of smaller grains compared to BTO and the reconstruction of the structural order caused by the incorporation of ions Sr^2+^ with a smaller ionic radius than Ba^2+^ into the lattice [24]. 

Because the ionic radii of Ba^2+^ and Sr^2+^ and the polarity of covalent bond are different (electronegativity 0.89 and 0.95, respectively), it should be expected that the competition between Ba-O and Sr-O bonds exists, which increases the structural disorder. This also depends on chemical stoichiometry and point defect chemistry, i.e., the formation of strontium and oxygen vacancies related to fluctuations in the composition, which is the result of the sintering process (a diffusion of Sr ions during synthesis). 

### 2.3. Application in Catalysis

Prior to the catalytic activity performance studies, the adsorption properties towards 4-NPh of the Ba_(1−x)_Sr_x_TiO_3_ series has been studied. The materials show a negligible adsorption (less than 2%) of 4-NPh after 180 min of contact.

The catalytic performance, kinetic properties and recyclability of the Ba_(1−x)_Sr_x_TiO_3_ perovskite series were examined in the reduction of 4-NPh to 4-APh in the presence of NaBH_4_. The reaction progress was controlled by UV–Vis, and a continuous decrease in the nitrophenolate peak at 400 nm with an increase in a new peak at 300 nm corresponding to 4-APh (Figure S1 in Supplementary Materials) was observed. Also, the presence of isosbestic points in the absorption spectra of the reacting mixture at different times provides clear evidence that the 4-NPh was selectively reduced to 4-APh [25]. In contrast, without adding any catalyst, but in the presence of NaBH_4_, the absorbance intensity and the intense yellow colour of 4-NPh is maintained for up to 5 h.

**Figure 5:**
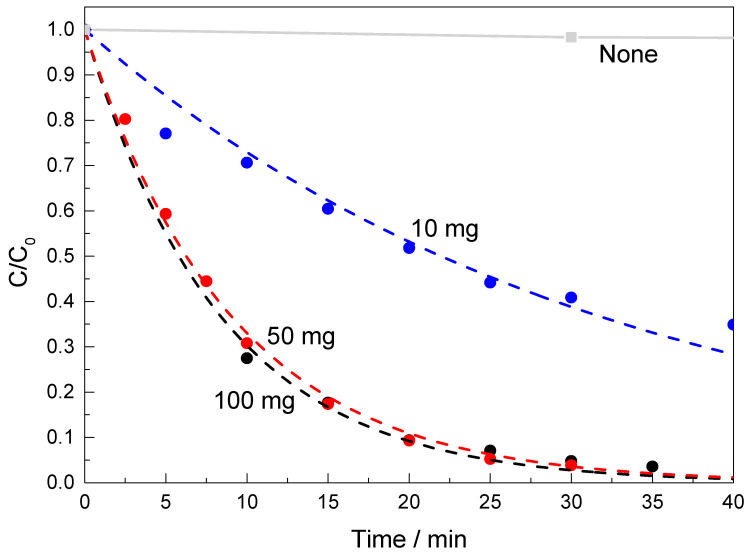
Catalytic reduction profiles of 4-NPh over BST_70, together with blank experiment (without catalysts); dashed lines are fitting curves to the pseudo-first-order model. Fixed operating conditions: c_4-NPh_ = 5 × 10^−5^ M, 30 mL, 56.7 mg of NaBH_4_.

Figure 5 shows the effect of the catalyst amount (10, 50 and 100 mg) on the reduction of 4-NPh to 4-APh when catalysed by BST_70; corresponding changes in UV–Vis spectra of 4-NPh aqueous solution are presented in Figure S1 in the Supplementary Materials. Increasing the catalyst dosage from 10 to 50 mg enhances the 4-NPh conversion from 83% to 99% and decreases the reaction time from 180 min to 30 min. In addition, increasing catalyst dosage from 10 to 50 mg increased the reaction rate from 3.16 × 10^−2^ min^−1^ to 1.11 × 10^−1^ min^−1^. However, the rate increased slightly when catalyst weight increased to 100 mg (1.19 × 10^−1^ min^−1^). Due to that, the optimum dosage of the catalyst was selected as 50 mg.

**Figure 6:**
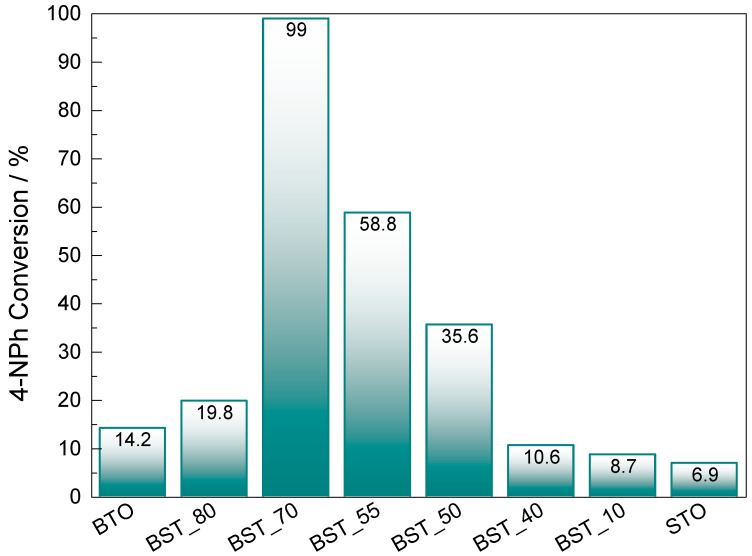
Catalyst effect on 4-NPh conversion in 90 min. Operating conditions: m_catalyst_ = 50 mg, c_4-NPh_ = 5 × 10^−5^ M, 30 mL and 56.7 mg of NaBH_4_.

The catalytic activity of the Ba_(1−x)_Sr_x_TiO_3_ perovskite series has been studied under optimized conditions. Corresponding comparisons of time-dependent UV–Vis spectra of 4-NPh solution are presented in Figure S2 in the Supplementary Materials. In the presence of the catalysts, the reduction reaction is initiated by the catalytic conversion of the 4-nitrophenolate solution to 4-APh (no induction time is needed), and complete substrate conversion occurs for the BST_70 catalyst in 40 min. The order of activity of the Ba_(1−x)_Sr_x_TiO_3_ perovskite series is as follow: BST_70 (99.0%) > BST_55 (58.8%) > BST_50 (35.6%) > BST_80 (19.8%) > BTO (14.2%) > BST_40 (10.6%) > BST_10 (8.7%) > STO (6.9%) (Figure 6). These results are far more promising than those obtained by Wang et al. [26], where the Nb_2_O_5_, KTaO_3_, NaTaO_3_ and H_2_Ta_2_O_6_ perovskites were not active in 30 min, or by Guo et al. [27], where BaTiO_4_ with Al-loaded particles led to 60% of 4-NPh conversion within 40 min, in the presence of NaBH_4_. Similarly, STO synthesized by the microwave-assisted hydrothermal method (180 °C, 30 min, power 1500 W) [15] was almost inactive in 4-NPh reduction using N_2_H_4_ as a hydrogen source (4-NPh conversion of 0.07, in 10 h); however, the same catalyst was highly active in nitrobenzene reduction under the same experimental conditions (total 4-NPh conversion in 60 min). 

**Figure 7:**
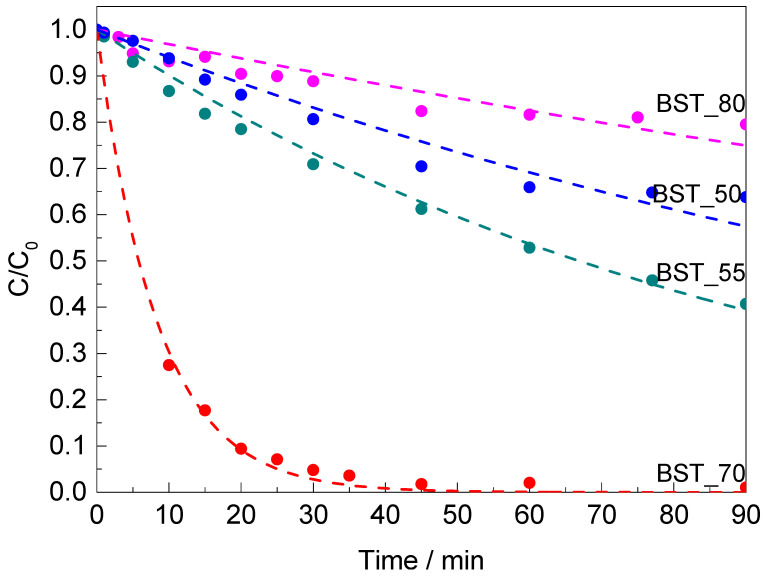
Catalytic profiles of 4-NPh reduction over the BST_50, BST_55, BST_70 and BST_80 catalysts; dashed lines are fitting curves to the pseudo-first-order model. Operating conditions: m_catalyst_ = 50 mg, c_4-NPh_ = 5 × 10^−5^ M, 30 mL, 56.7 mg of NaBH_4_.

To understand the kinetics of the catalytic reaction, the relationship between the reduction rate of 4-NPh and the reaction kinetic model is discussed. The studies were performed using BST_50, BST_55, SBT_70 and BST_80 catalysts, as other catalysts (STO, BST_20, BST_40 and BTO) show less than 20% of 4-NPh conversion in 90 min. Due to the excess of NaBH_4_, the reaction has been recognized as a pseudo-first-order reaction. The experimental data of corresponding catalytic profiles fitted to the pseudo-first-order model are presented in Figure 7. The results of the fitted parameters are summarized in Table 2. The pseudo−first-order rate constant (*k*) of 4-NPh reduction over BST_70 (*k* = 1.19 × 10^−1^ min^−1^) is 11.5, 19.3 and 37.1 times higher than those of BST_55 (*k* = 1.04 × 10^−2^ min^−1^), BST_50 (*k* = 6.16 × 10^−3^ min^−1^) and BST_80 (*k* = 3.21 × 10^−3^ min^−1^), respectively. 

The mechanism of catalytic reduction of 4-NPh in the presence of NaBH_4_ (reducing agent) has been the subject of many research studies. Two different routes have been proposed, namely direct via nitrosobenzene and N-phenylhydroxylamine intermediates or an indirect route that involves the condensation of nitrosobenzene and N-phenylhydroxylamine [28,29,30]. It was shown by Grzeschik et al. [29] that during the hydrolysis of NaBH_4_, the molecular hydrogen and species involved in the hydrolysis of BH_4_^−^ (H_3_B(OH)^−^, H_2_B(OH)_2_^−^, HB(OH)_3_^−^ and B(OH)_4_^−^) are formed. Both H_2_ and any of H_x_B(OH)_4−x_ species could generated the active [H]_ads_ species on the catalyst surface, which is a rate-limiting step. In general, the increase in dielectric constant favours the more polar site, and this normally means favouring the adduct formation. The high dielectric constants of BST_70 led to increasing the surface polarity and favours the adduct formation ([H]_ads_ species). The nucleophilic attack of hydride from [H]_ads_ to the nitrogen atom (4-NPh) yields the NHO_2_^−^ group and then is dehydroxylated to p-nitrosophenol and further converted into NHO^−^ and in water hydrolysis to the NH_2_O group (p-hydroxyaminophenol). It is further reduced to the NH_3_O^−^ group, and by dehydroxylation, the 4-APh is formed [31]. The specific molecular and electronic structure as well as dielectric properties of BST_70 change the electron density of the sample and could facilitate the generation of [H]_abs_ species on the catalyst’s surface and improve the reactivity of BST_70.

So far, 4-NPh catalytic reduction has been achieved mainly by noble-metal-based nanocatalysts [5,7] or lanthanide-containing perovskites [14]. Recently, studies using perovskite BiFeO_3_-phase and mullite-type Bi_2_Fe_4_O_9_-phase were published [32]. The oxides were prepared by using flame spray pyrolysis and tested as catalysts in reduction of 4-NPh in the presence of NaBH_4_. The reaction rate constants for these materials were 1.5 × 10^−2^ s^−1^ (9.1 × 10^−1^ min^−1^) and 0.23 × 10^−2^ s^−1^ (1.38 × 10^−1^ min^−1^), respectively, for BiFeO_3_ and Bi_2_Fe_4_O_9_. STO-Ag composites were used for reduction of 4-NPh in the presence of NaBH_4_ [33]. The best performance was observed for material containing 10% Ag particles, where complete reduction of 4-NPh was achieved within 7 min (*k* = 5.36 × 10^−1^ min^−1^). The material with 5% Ag showed enhanced conversion of the substrate (84.6% after 15 min, *k* = 1.17 × 10^−1^ min^−1^) vs. 51.6% after 40 min, *k* = 4.43 × 10^−2^ min^−1^ for 2% Ag@SrTiO_3_, and STO did not show obvious 4-NPh conversion in the same period [33]. STO decorated with Cu was tested in 4-NPh reduction and was inactive for 60 min [34]. Nano and bulk perovskite type SmFeO_3_ prepared by the microwave-assisted method were tested in hydrogenation of 4-NPh using isopropanol as a hydrogen source [35]. Both catalysts were active in 4-NPh reduction, and better results were found for the nanocatalyst (90% of 4-APh yield in 22 min) vs. bulk (38% of 4-APh yield in 35 min). The comparative results for the reduction of 4-NPh with BST-based catalysts, prepared using the one-step conventional method, indicate that the catalytic activity of BST_70 is better or at least comparable to the reported results.

**Figure 8:**
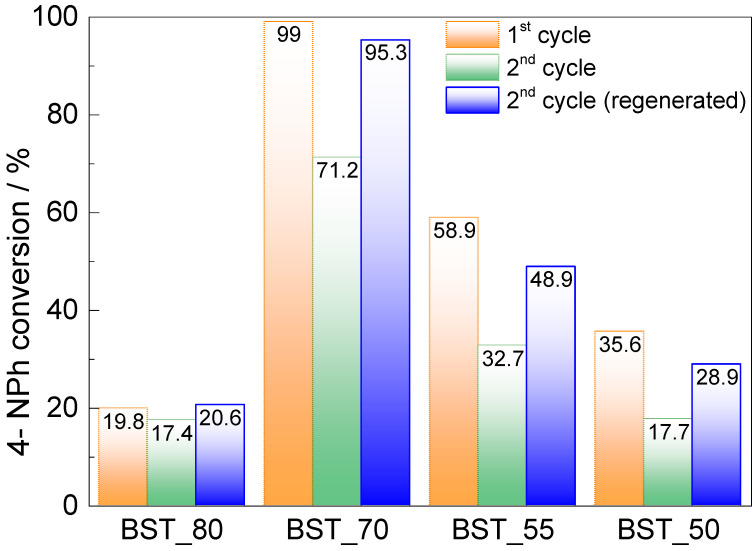
Comparison of 4-NPh conversion percentage, during its catalytic reduction, between the original (1st run), re-used without regeneration (2nd cycle), and regenerated catalysts (2nd cycle regenerated, after thermal regeneration: temp_max_ = 300 °C, 90 min).

The reusability of the most promising catalysts (BST_50, BST_55, BST_70 and BST_80), in which the substrate conversion ≥ 20% during 90 min, was studied in the consecutive catalytic runs, where 4-NPh reduction was carried out under the same conditions as the first reaction cycle: in water, in the presence of NaBH_4_ and with a reaction time of 90 min. After each cycle, the catalyst was removed from the reaction mixture by filtration. Then, the catalyst was washed with water and ethanol and dried at 90 °C overnight and used for the next cycle. The catalytic activity of the BST_80, BST_70, BST_55 and BST_50 slightly reduced in second cycle, respectively, by 2.4, 27.8, 26.2 and 17.9%. This result may be due to surface changes of the materials after contact (90 min) with NaBH_4_. However, the colour of the tested samples is maintained after the catalytic tests, and XRD patterns of the samples tested do not show obvious changes, i.e., neither new diffraction lines nor a broadening effect is observed. This suggests that the crystallinity of the sample remains unchanged, and no formation of amorphous disordered surface layer caused by perovskite interaction with NaBH_4_ takes place. It was reported that the formation of this amorphous layer significantly changed the activity of the perovskite catalyst (CaTiO_3_), due to electronic changes on the catalyst surface [36]. However, some ion diffusion or hydration of perovskites can occur during the catalytic process, which may lead to some phase transformation. The water molecules can diffuse into the perovskite lattice, leading to some changes in crystallinity of the samples and leading to a decrease in the activity of the catalysts. This effect was reported by Ma et al. [37], and the authors suggested that the process can be reversed by mild annealing of the perovskite sample. Thus, the catalyst regeneration experiment was performed, where the catalysts recovered after the first run were heated to 300 °C for 2 h. The catalytic properties of regenerated materials increased compared with the reuse assay. The regenerated catalysts exhibit enhanced catalytic activity compared to the second cycle, as shown in Figure 8, and the results are comparable to first use of the catalysts. These results suggest that the decrease in catalytic activity of the catalysts was due to sample hydration, and almost no damage effect of NaBH_4_ on the samples was performed.

## 3. Materials and Methods

### 3.1. Materials, Reagents and Solvents

All the reagents used during the experimental work were applied as received without further purification: barium carbonate (99.98%), strontium carbonate (99.5%), titanium oxide (≥99.9%), 4-nitrophenol (4-NPh, ≥99.5%), sodium borohydride (98%) and potassium bromide (≥99%, FTIR grade) were purchased from Merck (Rahway, NJ, USA) and Sigma-Aldrich. Ethanol (St. Louis, MO, USA) (99.8% pure p.a.) was obtained from Chempur (Karlsruhe, Germany). Ultrapure water (Millipore (Burlington, MA, USA), specific resistivity 18 MΩ cm) was used throughout the experiments.

### 3.2. Preparation of the Ba_(1−x)_Sr_x_TiO_3_ Ceramics

The preparation procedure of polycrystalline samples of Ba_(1−x)_Sr_x_TiO_3_ denoted as BTO, BST_80, BST_70, BST_55, BST_50, BST_40, BST_1 and STO, respectively, for x = 0.0, 0.2, 0.3, 0.45, 0.5, 0.6, 0.99 and 1.0 has been published elsewhere [38]. Briefly, the samples were prepared by a solid-phase reaction. The solids barium carbonate BaCO_3_, titanium oxide TiO_2_ and strontium carbonate SrCO_3_ depending on the desired material composition were ground in an agate ball mill in ethanol for 24 h (mixture homogenization). The dried material was calcined at the temperature of 1080 °C for 1–2 h. After the calcination, the powder was ground in ethanol, then cold-pressed (100 MPa) and sintered for 2–3 h with the aid of a conventional ceramic technology in the temperature range 1130–1230 °C, depending on the composition. 

### 3.3. Chemical and Physicochemical Characterization

The ceramics samples were digested in a two-step procedure using the Anton Paar Multiwave 3000 closed-system instrument (Anton Paar GmbH, Graz, Austria). For this purpose, 100 mg of the ceramic was weighed in a dedicated polytetrafluoroethylene tube. Then, 4 mL of HCl (35%), 2 mL of HNO_3_ (65%) and 2 mL of HF (47–51%) were added. The initial digestion was followed by complexation with saturated H_3_BO_3_ solution, which supports fluoride complexation and facilitates the dissolution of precipitated fluorides. Blanks were subjected to the same procedure. Next, after an appropriate dilution in deionized water, the samples were analysed for barium, strontium and titanium concentrations by an Inductively Coupled Plasma Optical Emission spectrometer ICP-OES (PlasmaQuant PQ 9000 Elite, Analytik Jena, Jena, Germany). The instrument settings are given in Supplementary Materials Table S1. The respective wavelengths of 455.403 nm for Ba, 407.771 nm for Sr and 334.941 for Ti were selected.

The microstructure tests of polycrystalline samples were carried out on fractures prepared in an identical way using a SEM Model Hitachi S4700 with a field emission and a Noran Vantage EDS system.

X-ray powder diffraction (XRD) patterns were collected using a PANalytical X’Pert Pro MPD diffractometer in the Bragg–Brentano reflection geometry with CuKα radiation from a sealed tube (Malvern Panalytical Ltd., Royston, UK). The apparatus operates in the range of 2θ = 5–90° with a step size of 0.0167°. 

The FTIR spectra were obtained in KBr pellets (Merck, spectroscopic grade), containing 2 wt.% material, 5:250 sample KBr ratio using a Jasco FT-IR 460 Plus spectrometer (Japan). All spectra were collected at room temperature, in the 400−4000 cm^−1^ range, using a resolution of 4 cm^−1^ and 32 scans. 

The dielectric measurements were made using a Novocontrol (NOVOCONTROL Technologies GmbH & Co. KG, Weilheim, Germany) Alpha High-Resolution Dielectric Analyzer in the temperature range from −100 to 250 °C and at frequency varying from 0.1 Hz to 10 MHz. Silver paint was used on the polished surfaces as electrodes. Nitrogen gas was used as a heating and cooling agent. 

Ultraviolet–visible (UV–Vis) spectra were registered on a Varian, Cary50Bio spectrophotometer (Palo Alto, CA, USA), in the range 500–200 nm, using quartz cell, path lengths l = 1 cm, 0.7 mL.

### 3.4. Catalytic Reduction of 4-NPh

In a typical experiment, 30 cm^3^ of a 5.0 × 10^−5^ mol·dm^−3^ stock solution of 4-NPh was transferred to a 50 cm^3^ round-bottom flask, and 56.7 mg of NaBH_4_ was added (0.015 mol·dm^−3^). The solution was subjected to constant stirring at room temperature in the dark. Upon addition of NaBH_4_, the yellow colour of the solution darkened due to the formation of phenolate ions (basic character of NaBH_4_, pH~10). After the addition of the desired amount of the Ba_(1−x)_Sr_x_TiO_3_ catalysts, the dark yellow colour of the solution progressively vanished, indicating the reduction of 4-NPh. Aliquots (0.7 mL) of reaction mixture were collected at fixed time intervals, centrifuged and analysed by UV–Vis. A control experiment was performed in the dark and in the absence of NaBH_4_ at pH 10 (addition of 100 mg of K_2_CO_3_ to 100 cm^3^ of 5.0 × 10^−5^ mol·dm^−3^ stock solution of 4-NPh).

The adsorption properties of the Ba_(1−x)_Sr_x_TiO_3_ for 4-NPh were evaluated by agitating 50 mg of the catalyst with 30 cm^3^ aqueous 4-NPh solution (5.0 × 10^−5^ mol·dm^−3^), at 500 rpm for 180 min.

Regeneration of the active catalysts. The highly active catalysts in the first reaction run with reduced activity in the second cycle were regenerated by mild thermal treatment. After the first catalytic run, the catalysts were separated by filtration and washed with water and ethanol and dried at 90 °C overnight. The dry samples were heated to 300 °C, and the solids were maintained at this temperature for 2 h. After cooling to ambient temperature, the catalytic activity of the thermally regenerated catalysts was tested under the same conditions as used in the first run.

## 4. Conclusions

In this work, the Ba_(1−x)_Sr_x_TiO_3_ perovskite series (0 < x < 1) were successfully prepared by cost-effective solid-phase reaction of respective precursors. All the materials presented high activity in valorization of toxic pollutants (4-NPh). The results from characterization methods revealed that increasing the strontium content decreases the BST samples’ unit cell parameters and grain size, which is related to conditions of the sintering process, influencing the maximum value of electrical permittivity and the phase transformation temperature and character. Regarding the catalytic activity, BST_70 presented the fastest kinetic profile in 4-NPh reduction due to the specific changes in the molecular and electronic structure. The thermo-regeneration data revealed that the materials recovered the catalytic activity obtained in the first cycle.

## Figures and Tables

**Table 1:** Barium, strontium and titanium contents [%] in the Ba_(1−x)_Sr_x_TiO_3_ (0 < x < 1) perovskites with the respective SE ^1^, *n* = 3. The theoretical element content values are given in parentheses.

Material	Found (Calculated)		
	Ba [%]	Sr [%]	Ti [%]
BTO	58.71 ± 0.17 (58.89)	ND ^2^	19.46 ± 1.15 (20.53)
BST_80	50.02 ± 0.52 (49.21)	7.90 ± 0.12 (7.85)	21.87 ± 0.52 (21.44)
BST_70	44.09 ± 0.53 (44.04)	12.45 ± 0.58 (12.04)	21.76 ± 0.17 (21.93)
BST_55	36.86 ± 0.58 (35.83)	19.11 ± 0.76 (18.70)	22.74 ± 0.12 (22.71)
BST_50	34.01 ± 1.24 (32.96)	21.42 ± 0.24 (21.03)	23.10 ± 0.13 (22.98)
BST_40	26.73 ± 1.15 (27.01)	26.59 ± 0.68 (25.85)	23.73 ± 0.16 (23.54)
STO	ND ^2^	47.83 ± 0.22 (47.75)	25.23 ± 0.35 (26.09)

^1^ Standard deviation of the mean of each element for each analysis (standard error) was calculated according to the standard formula ($SE=\frac{SD}{\sqrt{n}}$), where *SD* is standard deviation, n is the number of replicates. ^2^ ND—element concentration below the limit of detection.

**Table 2:** Fitting results of first-order kinetics model for different perovskite samples ^a^.

	Non-Linear Fitting of First-Order Kinetic Reaction		
Sample	*k* ^b^ (min^−1^)	R^2^	*k*’ ^c^ (min^−1^g^−1^)
BTO	2.71 × 10^−3^	0.95	5.42 × 10^−2^
BST_80	3.21 × 10^−3^	0.85	6.42 × 10^−2^
BST_70	1.19 × 10^−1^	0.99	2.38 × 10^0^
BST_55	1.04 × 10^−2^	0.99	2.08 × 10^−1^
BST_50	6.16 × 10^−3^	0.95	1.23 × 10^−1^
BST_40	1.11 × 10^−3^	0.96	2.22 × 10^−2^
BST_10	1.10 × 10^−3^	0.94	2.20 × 10^−2^
STO	1.09 × 10^−3^	0.98	2.18 × 10^−2^

^a^ Reaction conditions: c_4-NPh_ = 5 × 10^−5^ M, V_4-NPh_ = 30 mL, m_catalyst_ = 50 mg, m_NaBH4_ = 56.7 mg reaction time = 90 min; ^b^ apparent rate constant resulted from non-linear fitting of experimental data (*C* = C_0_ × e^(−*k*×t^) (*C* and *C_0_* are the 4-NPh concentrations of initial and at time t min); ^c^ calculated from *k’* = *k*/(the total weight of catalysts in g), where *k* is the first-order kinetic rate constant.

## Data Availability

Data are contained within the article and Supplementary Materials.

## Supplementary Materials

The following supporting information can be downloaded at: https://www.mdpi.com/article/10.3390/molecules29071416/s1, Figure S1: Change in UV–Vis spectrum of 4-NPh aqueous solution during the reduction of 4-NPh by NaBH_4_ in the presence of the BST_70 catalysts, catalyst dosage (A) m = 10 mg, (B) m = 50 mg, (C) m = 100 mg (initial 4-NPh concentration = 5.5 × 10^−5^ M, NaBH_4_ concentration = 0.05 M); Figure S2: Change in UV–Vis spectrum of 4-NPh solution during the reduction over (A) BTO, (B) BST_80, (C) BST_70, (D) BST_55, (E) BST_50, (F) BST_40, (G) BST_10 and (H) STO catalysts (experimental conditions: c4-NPh = 5.5 × 10^−5^ M, c_NaBH4_ = 0.05 M, catalyst dosage 1.7 mg mL^−1^, reaction time 90 min); Table S1: ICP-OES instrument settings for Ba, Sr and Ti determination.
