# Supplementary material for: Catalytic Transformation of Nitroarenes to Amines over Ba(1−x)SrxTiO3 (0 < x < 1) Perovskites in Water
Source: Molecules. 2024 Mar 22;29(7):1416. doi: 10.3390/molecules29071416 (PMC11012675; doi:10.3390/molecules29071416)

Catalytic Transformation of Nitroarenes to Amines over $Ba_{1-x}Sr_xTiO_3$ (0<x<1) Perovskites in Water

Iwona Kuźniarska-Biernacka [1,*], Barbara Garbarz-Glos [2,*], Elżbieta Skiba [3], Waldemar Maniukiewicz [3], Marta Monteiro 1, Wojciech Bąk [2], Dariusz Szydłowski [4] and Cristina Freire [1]

1 REQUIMTE/LAQV, Departamento de Química e Bioquímica, Faculdade de Ciencias, Universidade do Porto, Rua do Campo Alegre s/n, 4169-007 Porto, Portugal; marta_monteiro11@hotmail.com (M.M.); acfreire@fc.up.pt (C.F.)

2 Institute of Technology, University of the National Education Commission, Podchorążych 2, 30-084 Kraków, Poland; wojciech.bak@up.krakow.pl

3 Institute of General and Ecological Chemistry, Lodz University of Technology, Żeromskiego 116, 90-924 Łódź, Poland; elzbieta.skiba@p.lodz.pl (E.S.); waldemar.maniukiewicz@p.lodz.pl (W.M.)

4 Evidence Law and Forensic Technology, University of the National Education Commission, Podchorążych 2, 30-084 Kraków, Poland; dariusz.szydlowski@up.krakow.pl

* Correspondence: iwonakb@fc.up.pt (I.K.-B.); barbara.garbarz-glos@up.krakow.pl (B.G.-G.)

Table S1. ICP-OES instrument settings for Ba, Sr and Ti determination.

Parameter	Specification
Plasma power	1200 W
Plasma gas (Ar) flow	12 L/min
Auxiliary gas flow	0.5 L/min
Nebulizer gas flow	0.5 L/min
Pump rate	1.00 mL/min
Fast pump	4.00 mL/min
Measuring delay/Rinse time	45/15 s
Read time	3 s
Replicates	3

Figure S1. Change of UV–Vis spectrum of 4-NPh aqueous solution during the reduction of 4-NPh by $NaBH_4$ in the presence of the BST_70 catalysts, catalyst dosage (A) m= 10 mg, (B) m= 50 mg, (C) m=100mg (Initial 4-NPh concentration = 5.5×10^{-5} M, $NaBH_4$ concentration = 0.05 M).

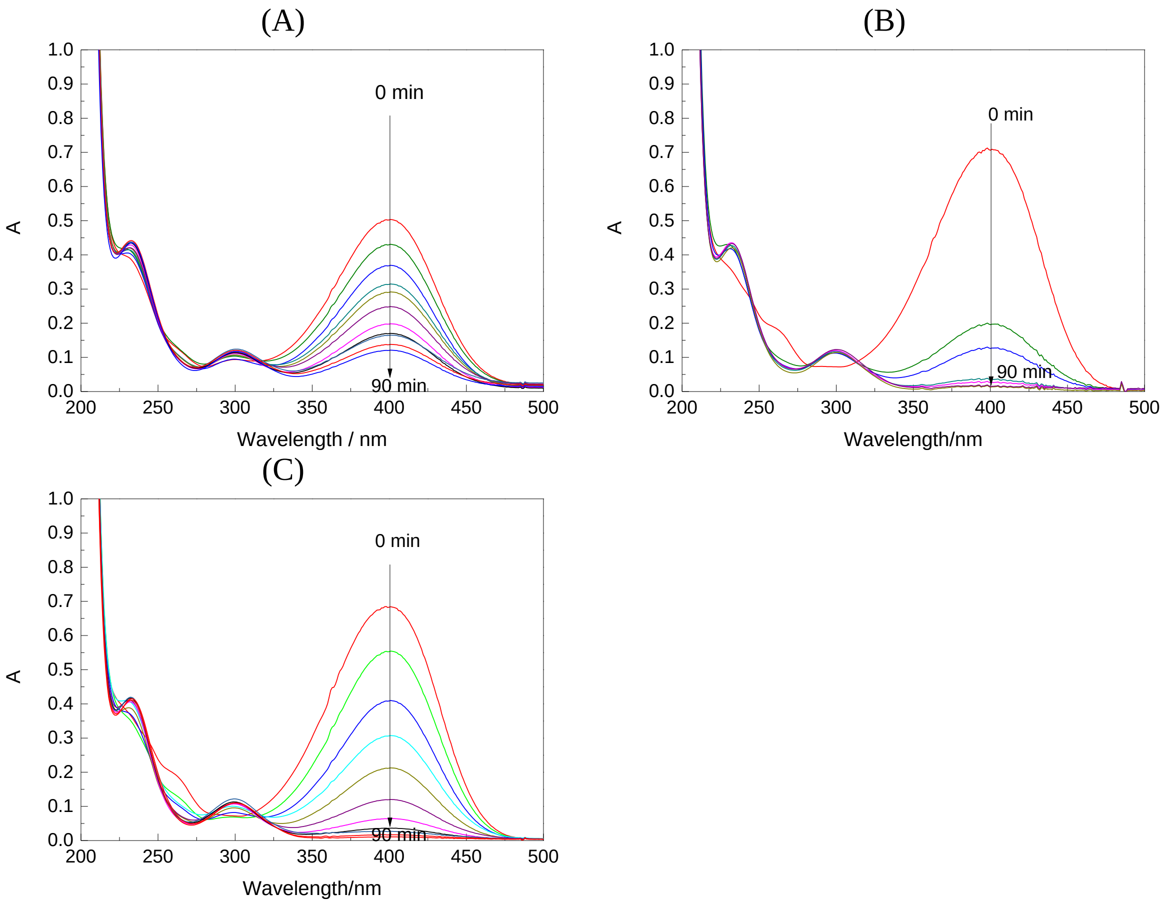

Figure S2. Change of UV–Vis spectrum of 4-NPh solution during the reduction over (A) BTO, (B) BST_80, (C) BST_70, (D) BST_55, (E) BST_50, (F) BST_40, (G) BST_10 and (H) STO catalysts, (experimental conditions: $c_{4\text{-NPh}} = 5.5\times10^{-5}$ M, $c_{NaBH4} = 0.05$ M, catalyst dosage 1.7 mg mL^{-1}, reaction time 90 min).

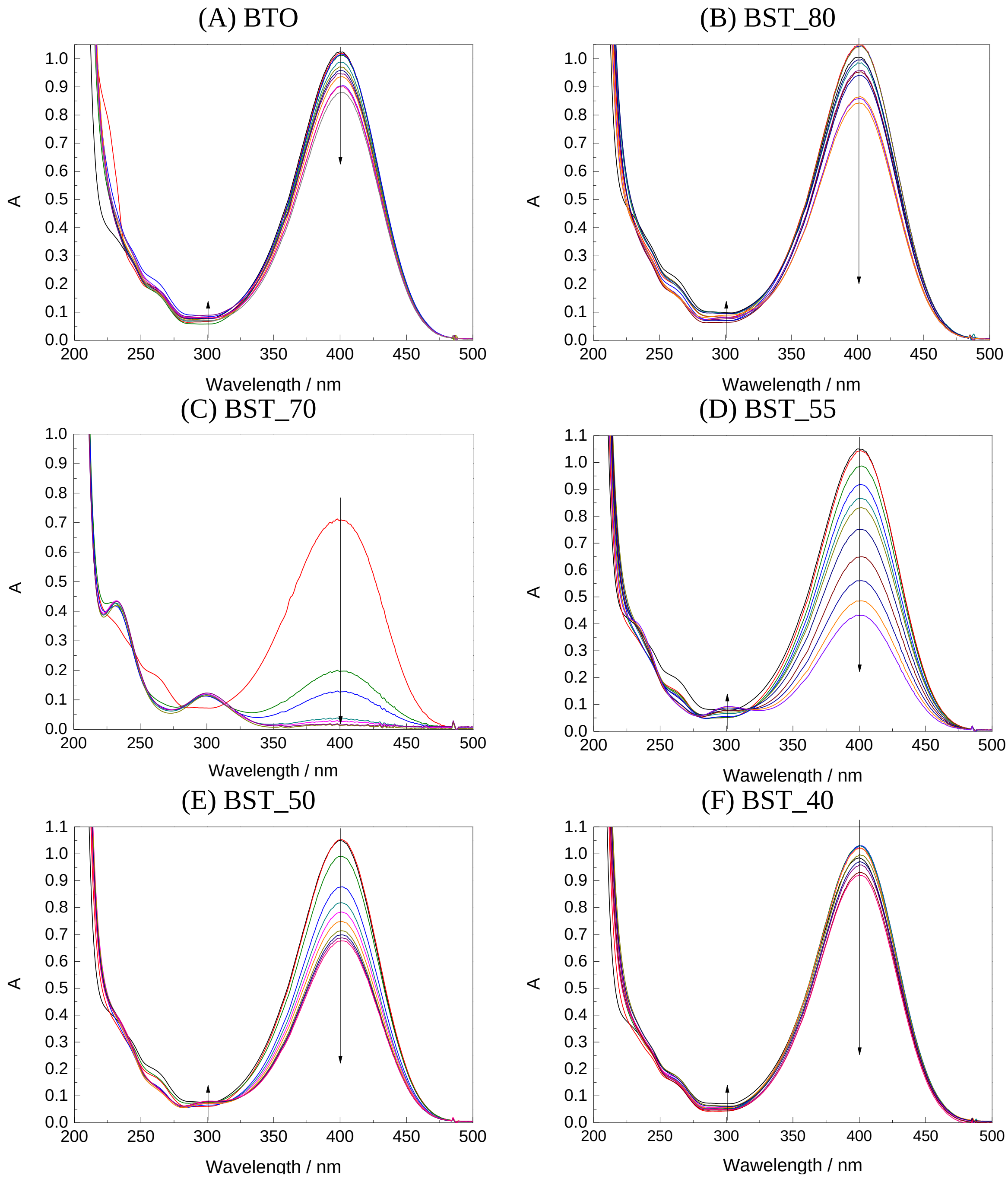

Figure S2 cont. Change of UV–Vis spectrum of 4-NPh solution during the reduction over (A) BTO, (B) BST_80, (C) BST_70, (D) BST_55, (E) BST_50, (F) BST_40, (G) BST_10 and (H) STO catalysts, (experimental conditions: $c_{4\text{-NPh}} = 5.5\times10^{-5}$ M, $c_{NaBH4} = 0.05$ M, catalyst dosage 1.7 mg mL^{-1}, reaction time 90 min).

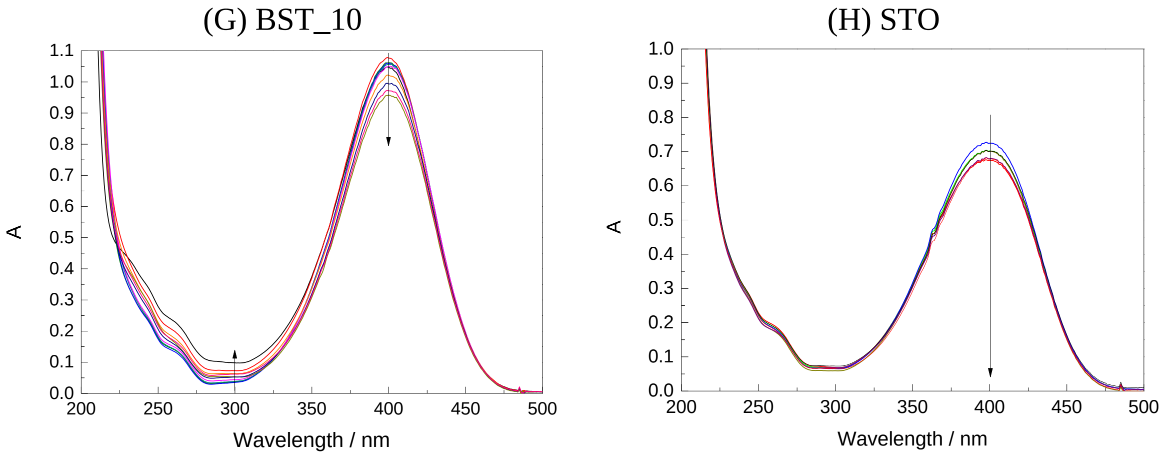

Supplement: Supplementary file 1 [file molecules-29-01416-s001.zip › molecules-2901390-supplementary.pdf]
